# Malaria vector control strategies in Pakistan: a scoping review

**DOI:** 10.1186/s12879-025-11347-x

**Published:** 2025-07-29

**Authors:** Hammad Atif Irshad, Hamzah Jehanzeb, Ayesha Yaseen, Umair Saleem, Muhammad Daniyal Javaid, Hafsa Khan Tareen, Muhammad Mukhtar, Henrique Silviera, Mohammad Asim Beg

**Affiliations:** 1 Medical College, Aga Khan University, Karachi, 74800 Pakistan; 2 Directorate of Malaria Control, Islamabad, Pakistan; 3 Global Health and Tropical Medicine (GHTM), Instituto de Higiene e Medicina Tropical (IHMT), Universidade NOVA de Lisboa (UNL), Lisbon, Portugal; 4 Section of Microbiology, Department of Pathology and Laboratory Medicine, Aga Khan University Hospital, Karachi, 74800 Pakistan

**Keywords:** Vector control, Malaria, Endemic, WHO

## Abstract

**Background:**

Malaria remains a significant public health concern in Pakistan due to its subtropical climate and diverse array of vectors, which contribute to periodic outbreaks and challenges in disease control. Recent outbreaks—particularly in 2022—along with the rising incidence of *Plasmodium falciparum* and growing resistance of *Anopheles* mosquito vector to existing control methods, highlight a critical gap in understanding the effectiveness of current malaria vector control strategies. This article is a scoping review of published literature on malaria prevention methods with a focus on World Health Organization (WHO) outlined interventions in the endemic region of the lower middle-income country, Pakistan.

**Methods:**

Relevant articles published in all languages before September 2023 were reviewed. All the articles were obtained from PubMed, Scopus, CINAHL, Embase and Google Scholar. Four independent reviewers performed the selection and characterization of articles based on defined inclusion criteria. The data collected were extracted and analyzed by province, vector, and vector control methods according to WHO recommendations.

**Results:**

A total of 46 articles reporting surveillance findings on vector control methods in Pakistan were found. Based on WHO recommendations, the reported strategies included insecticide-treated nets (ITN) (29.79%), indoor residual spraying (IRS) (29.79%), spatial spraying (12.77%), spatial/airborne repellents (4.26%), larval source management (4.26%) and house modifications (4.26%). In contrast to Khyber Pakhtunkhwa, which employs ITN (55%) as the primary vector control method, Punjab was found to use IRS as the main method of vector control (50%).

**Conclusion:**

This review highlights the current strategies for controlling malaria vectors and the strategies used in the past for outbreaks in Pakistan. This review identifies a notable increase in the use of insecticide-treated nets (ITNs) over time and highlights differences in the implementation of vector control strategies across provinces in Pakistan. Current practices and their contrast to WHO guidelines are illustrated. It helps us understand the need for improved research and development with precise reporting. These findings can serve as a reference for guiding policy decisions and improving malaria control efforts in endemic regions.

**Graphical Abstract:**

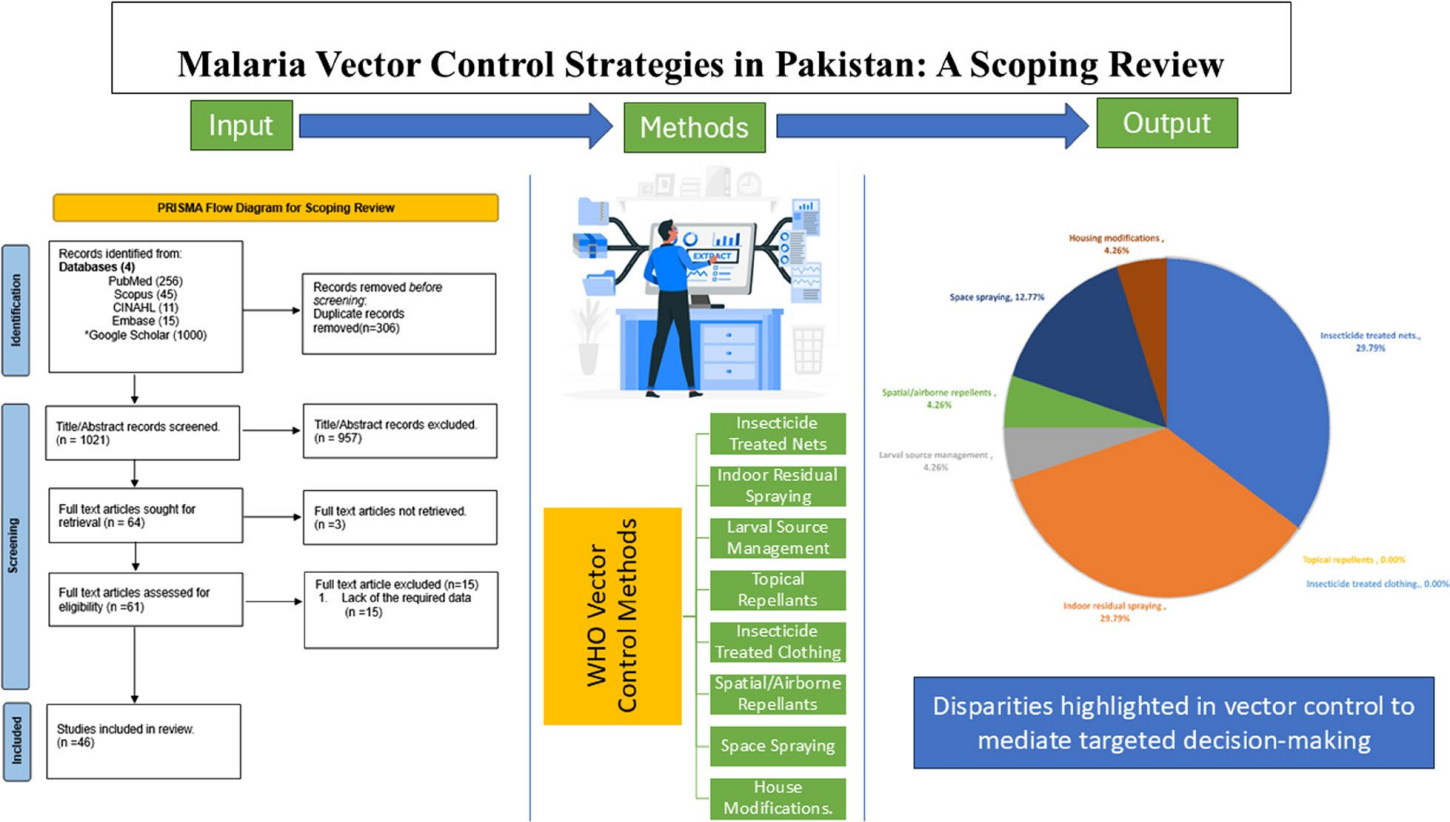

**Supplementary Information:**

The online version contains supplementary material available at 10.1186/s12879-025-11347-x.

## Introduction

The World Malaria Report for 2023 reveals an increasing trend of malaria cases from the preceding year reaching 249 million reported cases [1]. Malaria remains a major public health concern in South Asia with an estimated 5.2 million cases annually and approximately 8000 deaths [2].

Countries with subtropical climates, like Pakistan which we report on, have a concerning spectrum of vector-borne illnesses (VBDs), including leishmaniasis, dengue, malaria, chikungunya, and Crimean-Congo Hemorrhagic Fever (CCHF) [3]. In 2022, Pakistan experienced a significant surge in suspected malaria cases, exceeding 3.4 million, up from 2.6 million in 2021, marking the most severe outbreak in the last five decades [3].

Urgent attention to the issue is needed after the recent surge in the more lethal *Plasmodium falciparum* infections stated by the 2022 Directorate of Malaria meeting [4], accompanied by concern for chloroquine resistance persisting even 15 years after its discontinuation as treatment [5, 6]. Further debate also stems from Snow et al..’s findings indicating the reported prevalence of *P. falciparum* cases might be 50% higher than what WHO estimates, with almost one-third of the global incidence occurring outside the previously highlighted numbers from Africa [7].

Malaria’s unstable and seasonal spread through the bites of infected female Anopheles mosquitoes presents a multifaceted challenge across Pakistan’s 72 highly endemic districts, primarily situated in Balochistan, the Federally Administered Tribal Areas (FATA), Sindh, and Khyber Pakhtunkhwa (KPK) [3, 8]. Predominant species distribution constitutes 79.13% as *P. vivax*, with its difficult long-term clinical management, and *P. falciparum* at 16.29%, with its pharmacological resistance to common antimalarials [8].

While the 2022 World Malaria Report outlined some factors that play a role, such as a scarcity of resources and skilled human capital underscored by the gap in the amount invested and the funding needed [3, 9, 10], there appears to be more to the picture as we elaborate. The malaria epidemic in Pakistan results from a complex interplay of factors that we report on, encompassing natural disasters, urbanization, refugee camps, and an unbridled population growth [11, 12].

At the forefront of the global battle against malaria, the World Health Organization’s (WHO) Global Malaria Program (GMP) advocates for ITNs and IRS for at-risk populations. A substantial 78% reduction in clinical malaria cases was observed globally between 2000 and 2015 after widespread ITN and IRS scale-ups [13]. The World Health Assembly’s adoption of the “Global Technical Strategy for Malaria 2016–2030” [9] serves as a guide for measures to control vectors [14]. The report emphasized that strengthening malaria surveillance is fundamental to program planning and implementation and is a crucial factor for accelerating progress.

Our study aims to survey vector control data to help bridge this distribution gap and measure the effectiveness [9]. Certain limitations are unique to LMICs and may play a role in how effectively WHO guidelines are followed. For instance, ITN, despite being the mainstay of vector control in Pakistan, could not be distributed in 2021 due to COVID-19 pandemic-related disruptions in service [10]. This disruption may have had downstream consequences on vector population dynamics and disease transmission burden in subsequent seasons. Additionally, the catastrophic floods of 2022 exacerbated the situation, as evidenced by the alarming spike in confirmed cases [15].

This study’s significance in Pakistan might still be underscored by the country’s escalating malaria incidence which raises concerns about the implementation of vector control practices. Our work aims to bridge the gap in the literature on vector control for VBDs in Pakistan—a country burdened by a high malaria incidence but lacking substantial research.

## Methods

Our study was conducted in accordance with the Preferred Reporting Items for Systematic Reviews and Meta-Analyses extension for Scoping Reviews (PRIMA-ScR) [16].

### Search strategy and data sources

We used the following key terms: “Malaria AND “Pakistan” AND “Vector Control” OR “Prevention” OR “Eradication”. All possible word variations and medical subject headings (MeSH) terms were added to the search command.

The following databases were searched for articles: PubMed, Embase, CINAHL and Google Scholar. A reference snowballing technique was also utilized among topic-related articles to ensure the collection of the maximum number of articles. The literature search followed a scoping review methodology because there is limited published literature evaluating the use of WHO- ordained vector prevention methods in Pakistan.

### Study selection criteria

#### Inclusion criteria

Articles were included in this scoping review following the Population, Concept, Content (PCC) format. Therefore, the articles had to meet all three inclusion criteria below:

1. Population: Malaria Vectors in Pakistan.
2. Concept: Vector control strategies for malaria.
3. Content: Published articles describing clinical trials, case‒control studies, cross-sectional studies, and other epidemiological studies were included in this review. The interventions in the studies that were considered had the following outcomes: they all had an effect or impact on the malaria burden in the community or field site, led to a reduction in malaria transmission in the community; and were affordable and hence had the potential to be sustainable in low-income settings.

#### Exclusion criteria

We excluded all studies in which the study area was not Pakistan. Furthermore, studies assessing vector-borne diseases other than malaria, addressing epidemiology, burden of disease, or reviews on the topic of malaria were excluded. Studies on the seroprevalence and molecular biochemistry of malaria transmission and associated strains were also excluded.

Articles meeting the above criteria were included regardless of their publication year, methodology or scope. Articles that focused on interventions that were not primarily aimed at malaria prevention were excluded.

Rayyan AI software was used to help organize and review all the relevant literature. An initial screening was performed based on the inclusion criteria (HI). Abstract and full-text screening of the articles was performed by three independent reviewers (AY, HT, MDJ), and any conflicts were resolved by HI.

After the relevant studies were shortlisted, data extraction was performed manually by four reviewers (AY, HJ, US, MDJ) to obtain all the relevant data.

### Data extraction, summary, and analysis

The following extraction grid was created in Microsoft Excel for each of the WHO ordained vector control methods: ITN, IRS, Larval Source Management, Topical Repellants, Insecticide Treated Clothing, Spatial/Airborne Repellants, Space Spraying, and House Modifications.

Data extraction was performed by three reviewers independently to ensure consistency, focusing on study type, location, year, WHO ordained vector intervention details, and outcomes derived through those interventions.

The details of the interventions in the included studies were combined as a narrative review. For qualitative information, the results for each identified theme were tabulated. No software or formal qualitative synthesis tools were used; rather, thematic analysis was conducted manually based on patterns emerging from the data. Judgments on the methodological quality of the included studies were not made, since most were cross-sectional descriptive surveys, surveillance reports or retrospective charts. Rather, analyses were stratified based on interventions identified by province and year.

Since the objective of this review was to scope and map the literature on malaria vector control strategies, the WHO-outlined vector control methods used in Pakistan were not used to assess the methodological quality or bias risk of the included studies. This is consistent with the guidance on conducting scoping reviews. However, it is important to underscore that the included studies -given that they are primarily cross-sectional surveys, surveillance reports and retrospective charts– are prone to selection and reporting biases.

## Results

### Study selection (Fig. 1)

**Fig. 1:**
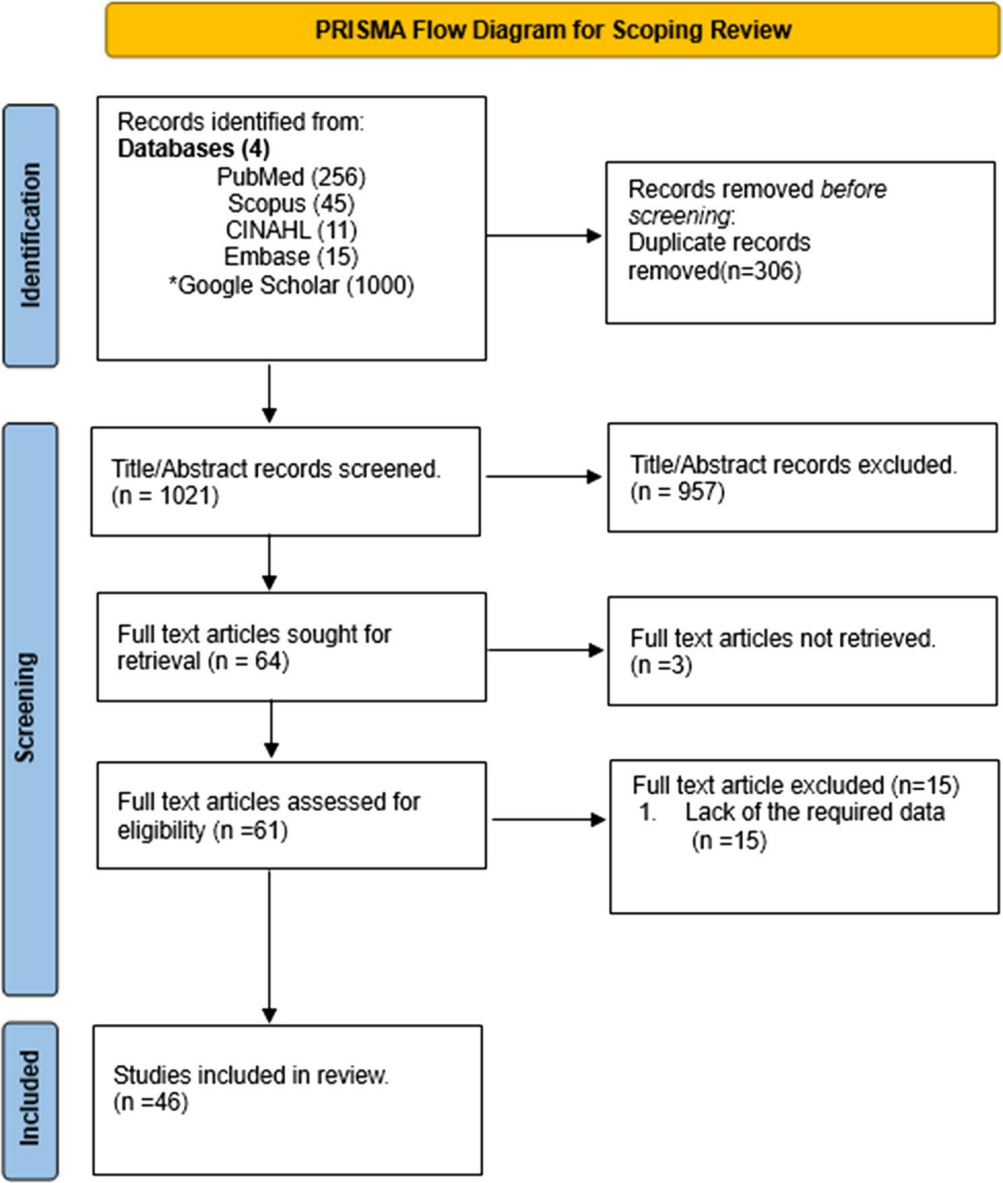
PRISMA flow diagram. * A Google Scholar search was performed using Publish or Perish software, restricting the results to 1000 articles published between 2000 and 2023. “n” is the number of articles

A comprehensive search across 5 databases yielded a total of 1327 publications. After screening the titles and abstracts and removing duplicate records, 61 articles were chosen for an in-depth evaluation of their full texts. Ultimately, 46 papers met the eligibility criteria and were included in the study.

### Characteristics of included studies

The selected studies were predominantly from KPK/FATA (23/46) and Punjab (18/46). There were only 3 studies, each from Sindh and AJK, and none were from Baluchistan or Gilgit Baltistan. Additionally, one study, a letter to the editor, discussed vector control in Pakistan in general instead of in a particular province. Almost all the studies were cross-sectional (23/46) or experimental (19/46) studies, as shown in Supplementary Table 1. Additionally, we included 3 letters to the editor and one audit of a malaria control project in KPK. Almost half of the studies (22/46) were conducted from the 2010 onward, with a lesser number of studies being from the 199s (11/46) and 2000s (9/46). Additionally, there were 4 studies from the 1980s–1970s.

### Vector characteristics (Table 1)

**Table 1:** Study characteristics

Study Author Last Name	Year	Province	Agent	Vector
Reisen et al. [17]	1978	Punjab	NR	*An.culicifacies* *An.stephensi* *An.subpictus* *An.culicifacies*
de Zulueta et al. [18]	1980	Punjab	NR	NR
Nasir et al. [19]	1982	Punjab	NR	NR
Reisen et al. [20]	1986	Punjab	NR	*An.culicifacies* *An.stephensi* *An.subpictus* *An.annularis* *An.pulcherrimus*
Chester et al. [21]	1992	Punjab	NR	NR
Bouma et al. [22]	1995	KPK	NR	NR
Hewitt et al. [23]	1995	KPK	NR	*An.stephensi*
Rowland et al. [24]	1996	KPK	*P. falciparum* *P. vivax*	NR
Hewitt et al. [25]	1996	KPK	NR	*An.stephensi*
Shah et al. [26]	1997	KPK	*P. falciparum*	NR
Donnelly et al. [27]	1997	Punjab	NR	NR
Rowland et al. [28]	1997	KPK	*P. vivax*	NR
Rowland et al. [29]	1997	KPK	*P. falciparum* *P. vivax*	NR
Rowland et al. [30]	1999	KPK	NR	*An. stephensi*
Hewitt et al. [31]	1999	KPK	NR	NR
Rowland et al.[32]	2001	KPK	*P. vivax* *P. falciparum*	*An. stephensi* *An. culicifacies*
Rowland et al. [33]	2001	KPK	NR	NR
Rowland et al. [34]	2001	Punjab	NR	*An.culicifacies* *An.stephensi*
Graham et al. [35]	2002	KPK	NR	*An.stephensi* *An.pulcherrimus* *An.nigerrimus*
Herrel et al. [36]	2004	Punjab	NR	*An.subpictus s.1.1* *An.stephensi* *An.culicifacies s.1.* *An.pulcherrimus* *An.peditaeniatus*
Graham et al. [37]	2004	KPK	NR	*An.stephensi*
Graham et al. [38]	2005	KPK	NR	*An. stephensi* *An. subpictus* *An. nigerrimus* *An. pulcherrimus*
Asif et al. [39]	2008	KPK	*P. falciparum*	NR
Leslie et al. [40]	2009	FATA	*P. falciparum*	NR
Rathor et al. [41]	2013	Punjab	NR	*An. stephensi* *An. culicifacies* *An. subpictus*
Nazir et al. [42]	2013	Punjab	NR	*An. stephensi*
Malik et al. [43]	2013	Punjab	NR	*An. culicifacies* *An. stephensi*
Mehmood et al. [44]	2013	Punjab	NR	*An. culicifacies* *An. stephensi*
Haidera et al. [45]	2013	Punjab	NR	NR
Batool et al. [46]	2014	AJK	NR	*An. culicifacies* *An. stephensi*
Rana et al. [47]	2014	Punjab	NR	*An.stephensi* *An.culicifacies* *An.fluviatilis* *An.superpictus* *An.subpictus*
Hammad et al. [48]	2015	Sindh	NR	*An.subpictus*
Hammad et al. [49]	2015	Sindh	NR	*An.subpictus*
Chaccour et al. [50]	2016	Pakistan	NR	NR
Karim et al. [51]	2016	KPK	*P. vivax* *P. falciparum*	NR
Howard et al. [52]	2017	KPK	*P. vivax* *P. falciparum*	NR
Younis et al. [53]	2017	Punjab	NR	NR
Howard et al. [52]	2017	KPK	*P. falciparum* *P vivax* Mixed infections	NR
Qureshi et al. [54]	2019	Punjab	*P. vivax* *P. falciparum*	NR
Jahan et al. [55]	2019	KPK	*P. vivax* *P. falciparum*	NR
Naeem et al. [56]	2019	Punjab	NR	*An.subpictus*
Farooqi et al. [57]	2019	FATA	*P. vivax* *P. malariae*	*An. stephensi* *An. culicifacies*
Qureshi et al. [58]	2020	KPK	*P. vivax* *P. falciparum*	NR
Kumar et al. [59]	2020	Sindh	*P. vivax* *P. falciparum* Mixed infections	NR
Karim et al. [60]	2021	KPK	*P. vivax* *P. falciparum*	NR
Mohsin et al. [61]	2021	Punjab	NR	*An. stephensi* *An. culicifacies* *An. subpictus* *An. annularis* *An. fluviaitlis* *An. pulcherimus*

Twenty-four studies reported the species of the vector being studied. Twelve of these studies were from Punjab, 9 from KPK, 2 from Sindh, and 1 from AJK. The most represented species were *Anopheles stephensi*, reported by 19 studies, followed by *An. culicifacies* (12/24) and *An. subpictus* (10/24). Additionally, 7 studies reported other species, such as *An. nigerrimus*, *An. pulcherrimus*, and *An. annularis*. In Punjab, *An. stephensi* was the most commonly reported vector (10/12), followed by *An. culicifacies* (9/12) and *An. subpictus* (7/12). Similarly, in KPK, the vector most commonly reported was *An. stephensi*, with all 9 studies from the province reporting this vector, followed by *An. culifacies* (2/9) and *An. subpictus* (1/9). Of the 2 studies from Sindh, both reported only *An. subpictus*. A single study from AJK reported both *An. stephensi* and *An. cululifacies*.

### Vector control methods for anopheles mosquitoes

**Fig. 2:**
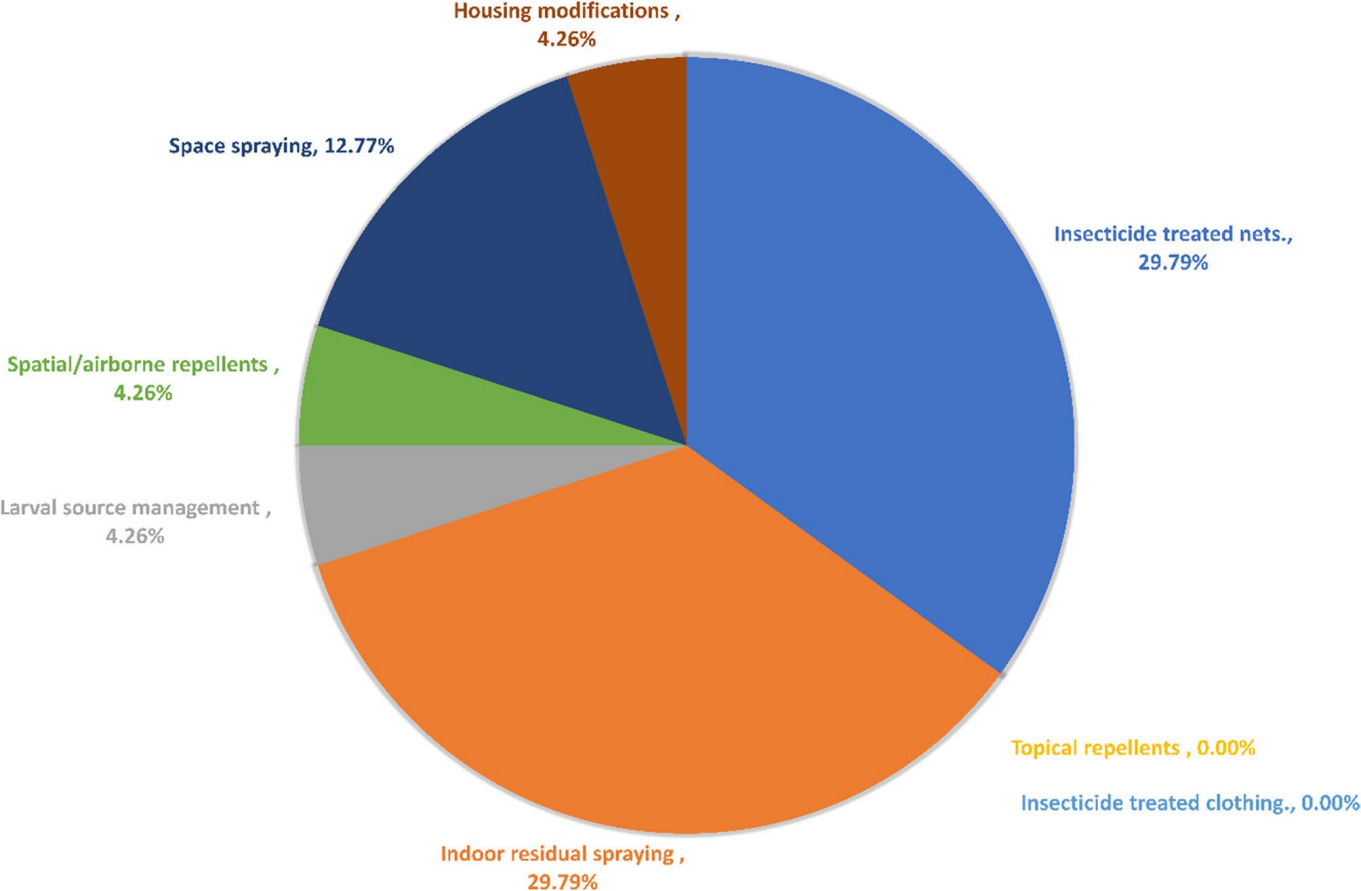
Percentage of studies showing vector control interventions in Pakistan

Our study revealed that the primary vector control methods implemented nationwide in Pakistan were ITN and IRS, which were both reported by 14 out of the total 46 studies (29.79%). Space spraying constituted 12.77%, while larval source management, housing modifications, and spatial/airborne repellents each accounted for 4.26%. There were no studies reporting on the use of topical repellents or insecticide-treated clothing. Our findings are depicted in Fig. 2.

### Vector control methods in different provinces

**Fig. 3:**
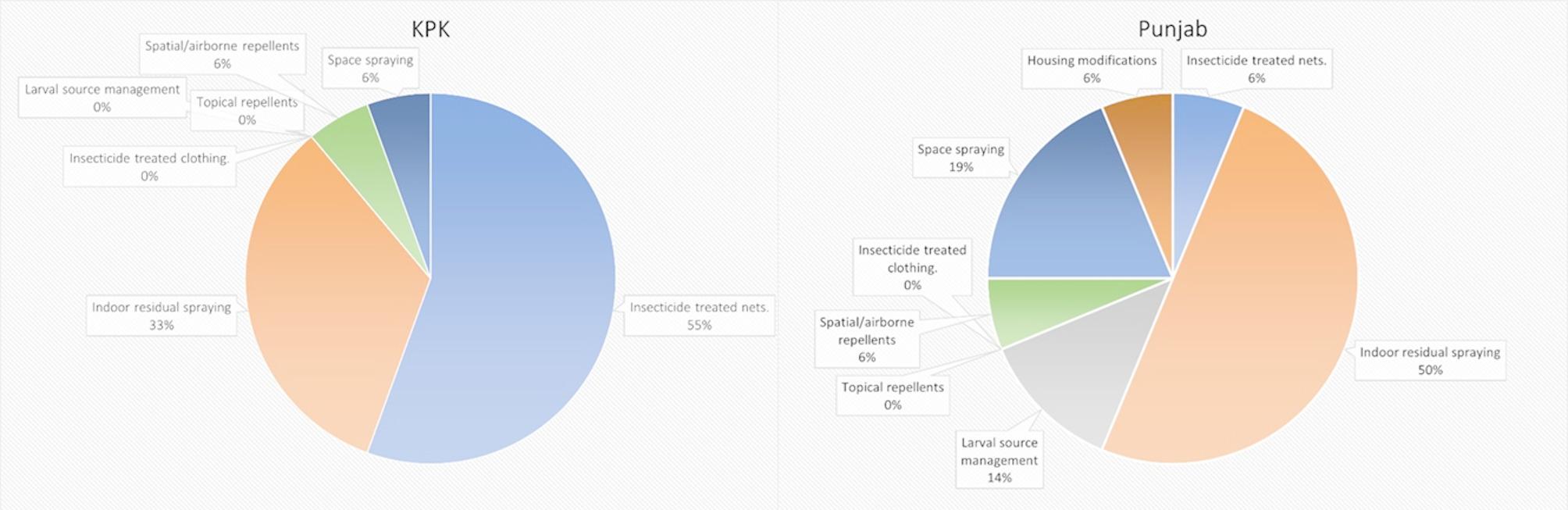
Vector Control Methods for KPK and Punjab

Stratifying the vector control methods by province, as shown in Fig. 3, revealed that the most commonly reported control strategies in Punjab were IRS (50%), space spraying (19%), and larval source management (14%). In contrast, 55% of all vector control strategies used in KPK were ITNs– compared to just 6% in Punjab. This was followed by IRS (33%), space spraying (6%), and spatial/airborne repellents (6%). Moreover, no studies from KPK reported the use of larval source management or housing modifications, whereas these methods were evaluated in 14% and 6% of studies from Punjab, respectively.

### Vector control methods by year

**Fig. 4:**
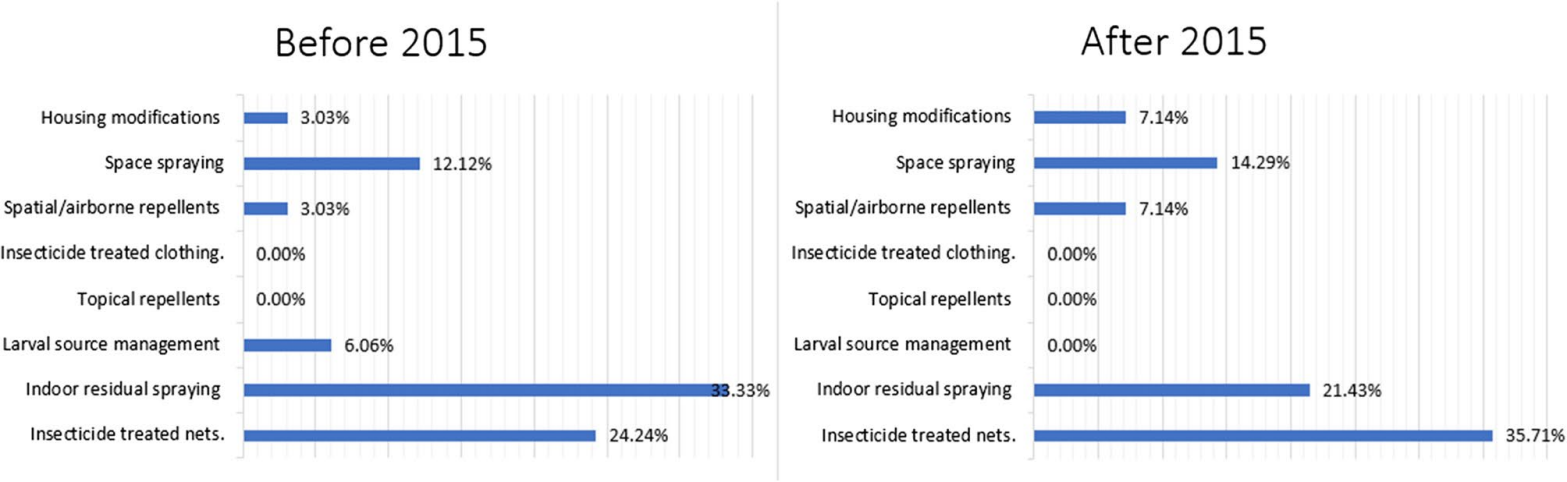
Vector control methods before and after 2015

Stratification was further performed before and after 2015 to determine the impact of the 2015 World Malaria Summit recommendations on their implementation. The summit set a 2025 milestone for a 75% reduction in malaria cases by 2025 for the Eastern Mediterranean Region [62]. There was a marked difference in the vector control methods employed by studies prior to 2015 and thereafter, as shown in Fig. 4. Before 2015, the most common method was IRS, followed by ITN, whereas after 2015, the most common method was ITN followed by IRS.There was a relative increase in the use of vector control methods after 2015 in the share of studies analyzing housing modifications (3.03–7.14%), space spraying (12.12–14.25%), spatial/airborne repellents (3.03–7.14%), and ITN (24.24–35.71%), whereas there was a decrease in those studying IRS (33.33–21.43%). In fact, there were no studies on larval source management post-2015.

## Discussion

Our scoping review is the first to evaluate all the present literature on malaria to assess the implementation of WHO-recommended vector control methods based on published literature [63]. Data from our study aligns with the WHO Malaria reports that ITN and IRS are two of the most common vector control measures. We, however, noted that all other WHO recommended measures such as topical repellents and insecticide-treated clothing are less frequently or sometimes, in our case, never used and may not reflect a true picture.

The 2017–2018 Demographic and Health Survey of Pakistan, the latest of the series, revealed that, nationally, only 23% of households owned a mosquito net. However, only 4% of these were ITNs. As for access to these ITNs, which was estimated via use of the net in the household the night before the survey, the estimate drops further to 2%. Notably, rural areas had greater availability (5%) than urban areas (2%), this disparity could be explained by mass distribution strategies primarily targeting rural areas such as KPK, FATA, Balochistan ITN support in Pakistan, primarily backed by The Global Fund 2009, initially targeted high-risk populations. Overtime ITN intervention further expanded to universally cover rural populations in selected districts with a mass distribution campaign strategy in 2018 [64]. Among the provinces, FATA reported the highest percentage (56%), followed by Balochistan (37.7%), Sindh (22.1%), and KPK (15%) [65]. 

IRS (29.8%) was the second most reported strategy. The effectiveness of the IRS as an intervention was initially shown during the Global Malaria Eradication Campaign (GMEP, 1955–1969), when the number of people at risk worldwide was reduced by 700 million using DDT in conjunction with case management, environmental management, and housing improvements [63, 66]. As a result of the GMEP, malaria was eliminated in 37 countries by 1978(25–27). According to the latest data, there are 27 approved products for IRSs and 26 for ITNs [67]. Pakistan has one of the best quality assurance protocols, which mandates the use of WHO-recommended pesticides and equipment for vector control and states that local pesticide manufacturers must document the origin of active and inert ingredients [67].

Commonly used insecticides in Pakistan are pyrethroids such as deltamethrin. Other classes of insecticides used include carbamates (e.g., bendiocarb, propoxur), organophosphates (e.g., pirimiphos-methyl), and historically, organochlorines like DDT, which share a similar target site with pyrethroids [65]. It is important to note that the IRS offers a possible alternative to pyrethroid resistant settings where ITNs are still the mainstay of treatment since IRS possesses the advantage of being able to make use of a much wider range of insecticide products in comparison to ITNs, for which pyrethroids are the only class of insecticide currently used [68]. Additionally, they may be added as an adjunct to ITNs in highly endemic, high burden regions which in the context of Pakistan would be regions of monsoon flooding and refugee camps. It is for this reason that the IRS method makes up the second largest share of intervention techniques employed. Despite the use of rigorous protocols and relative importance of IRS use in specific settings, results from our study show that these protocols were used less than were ITNs. This decline in its usage could largely be attributed to costs of maintaining operations, labor costs as well as transportation and storage challenges [69].

In our scope of the literature, there was no mention of insecticide-treated clothing or topical repellents. A possible reason for the lack of topical repellents is that previous studies from several malaria-endemic areas, such as Southeast Asia and South America, found that the use of repellents along with ITNs was insignificant compared to the use of ITNs alone making allocation of resources towards topical repellents a less feasible solution when ITNs alone can be attributed to significant decreases in malaria incidence [70–72].It is also important to note that the mention of repellents in vector control guidelines has only been made and of those that were mentioned, only WHO prequalified products such as N, N-Diethyl-meta-toluamide (DEET), hydroxyethyl isobutyl piperidine carboxylate (Icaridin), and ethyl butylacetylaminopropionate (IR3535) are recommended for use making any vector control strategies around based around repellent use incredibly challenging.

Another factor for lack of repellent use could be attributed to the repetitive nature of its application and the cost of the intervention, which in a resource limited setting, may not be the most feasible. Ziauddin et al. demonstrated a lack of awareness of mosquitoes as vectors and availability of anti-mosquito sprays in rural areas of Pakistan compared to urban areas (59% vs. 92%) [73]. DEET (di-ethyl 3-methyl benzamide) soap was compared to a placebo lotion in Afghan refugee households in Pakistan. The results for vivax malaria had no considerable difference although the true intervention effect may be masked by relapse infections in the endemic area. The repellent was received properly, and no side effects were reported [74].

Given this distinction, Pakistan’s public health interventions primarily prioritize IRSs and ITNs rather than investing extensively in outdoor-focused solutions such as insecticide-treated clothing. This emphasis on indoor transmission may contribute to the underdevelopment and limited adoption of insecticide-treated clothing within Pakistan’s public health strategies.

However, further research is needed to evaluate the potential effectiveness of treated clothing in Pakistan’s context. If proven effective, exploring engineering solutions such as promoting thick and thin layered clothing for different seasons could improve pan seasonal adherence and efficacy [75, 76].

It is important to note that according to the WHO, IRSs and ITNs are considered core interventions for malaria control, while LSMs are classified as supplementary interventions [13, 66]. Other interventions, such as repellents and cloth treatment, are categorized as community-owned interventions rather than nationally recommended interventions. This classification is due to the very limited efficacy of these treatments and other challenges such as the cost of the intervention, proportions of the global funding allocated to them as well as distributive and reapplication challenges for interventions such as topical repellents. Consequently, these interventions cannot be utilized for the evaluation of program efficacy and coverage.

Disparities regarding the application of vector control methods were also found in provinces of Pakistan in our scoping review. This could be credited to the differences in vector burden in some provinces, such as KPK and Punjab. Our findings also indicate the increased use of WHO-recommended strategies after the global malaria assembly was established in 2015, revealing that resolution does have an effect. However, at the same time, reinforcement of these measures is crucial to ensure long-term adherence.

To lower the chance of malaria transmission ITNs are frequently utilized and have been a component of global malaria control policies since the mid-1990s [77]. The results of our study revealed that only 30% of respondents from Pakistan reported using ITNs, 55% being the most reported method in KPK, but only 6% use in Punjab. This makes much greater use of these methods compared to other WHO-recommended vector controls.

Initiatives within countries are limited by their setting and available resources [78]. The extent of adherence to the WHO recommendations is likely due to Pakistan’s resource limitations. However, Pakistan must also improve its strategic approach while being cognizant of its existing resources. We recommend that while ITNs and IRS should remain the cornerstone of Pakistan’s National Malaria Vector Control Strategy, it is imperative to explore supplementary interventions, such as repellents and insecticide-treated clothing, particularly during monsoon floods and for populations in transient refugee settings. It is worth highlighting that the effective allocation of these limited resources hinges on precise, long-term spatiotemporal assessments of malaria burden in high-risk areas, enabling targeted interventions to maximize impact. For instance, to achieve the strategic goal of zero autochthonous malaria transmission by 2020, Iran’s national key control prevention policies successfully integrated interventions such as bed nets, indoor spraying, free malaria diagnosis, active or passive case recognition, disease treatment, and source reduction [79]. Such an interdisciplinary integration is imperative for Pakistan if we are to achieve the goals outlines in the GTS for Malaria.

### Limitations

This research has several limitations worth mentioning. This review primarily relies on published literature, which may exclude insights from unpublished sources. However, this approach ensures a rigorous and verifiable analysis based on peer-reviewed data. The authors, while conducting the study, faced challenges in finding published data on the coverage and efficacy of vector control interventions. The dearth of information on this subject is noteworthy, even though scoping reviews are not intended to offer comprehensive efficacy summaries. It draws attention to a gap in the existing literature that may help guide future investigations and choices about vector control methods in Pakistan. This constraint raises the possibility that more research is necessary to determine the efficacy of these measures, which might help shape the nation’s vector control strategy and point out knowledge gaps. In addition, there was an underrepresentation from some provinces in Pakistan such as Balochistan and Gilgit-Baltistan which should be addressed in future cohorts.

Second, some of the relevant literature was not published in PubMed-indexed journals or was in journals with lower impact factors. Regardless, the study has publication bias has online available literature was utilized. However, we did not explicitly assess the quality of the studies included due to the diverse study designs and methodologies employed. This lack of a standardized quality assessment means we cannot conclusively determine the quality of all the studies included. Additionally, there was no single standardized method for measuring the effectiveness of vector control strategies for malaria, which further complicates direct comparisons across studies.

### Recommendations

Thorough documentation and consequent publication are essential for improving research reporting on malaria vector control strategies in Pakistan and public awareness, respectively. Extensive documentation of intervention strategies—such as the use of insecticides, bed nets, and larval control techniques—should be provided by researchers. However, it is equally important to emphasize the documentation of surveillance, monitoring, and evaluation (M&E) data during program implementation, which is often underreported. This data is crucial for assessing the real-world effectiveness of these strategies and for informing future interventions.

To enable replication and validation, specific methods, such as data collection processes and sample strategies, need to be identified. Contextual elements influencing vector populations, such as geographic location and seasonal fluctuations, should also be included in reporting. For proper interpretation, limitations and biases must be disclosed in a transparent manner. The adoption of standardized reporting formats, such as STROBE for observational research and CONSORT for trials, can improve study clarity and comparability and eventually lead to progress in Pakistan’s efforts to control malaria.

The GTS strategy for 2021–2030 clearly states that countries where malaria is endemic need to harness innovations and increasingly engage in basic, clinical and implementation research. Implementation research will be fundamental to optimizing impact and cost − effectiveness and facilitating rapid uptake in populations at risk [9].

## Conclusion

Our scoping review provides an overview of the current literature on malaria vector control strategies in Pakistan, primarily focusing on published studies. It aims to summarize the available evidence on different interventions. Overall, our findings show differences in adherence to WHO recommendations, but our baseline assessment is relevant for implementing any improvement. Notably, studies showing an increase in certain interventions, alongside a decrease in others, suggest a shift in importance given to strategies in malaria control. This shift, particularly following the Malaria Assembly in 2015, indicates a growing recognition of the importance of specific strategies, which shows promise for future improvements in vector control efforts. Although several unpublished reports exist for malaria control, the publication of these reports is crucial for awareness. Through awareness and policy change, improved vector control measures in Pakistan present a unique opportunity to help Pakistan, one of the many regions affected by malaria, achieve its malaria control goals.

## Supplementary Information

Supplementary Material 1: Table 1. Study Characteristics (Included Articles=46).

## Data Availability

Available upon reasonable request from the corresponding author.

## Abbreviations

GMP: Global Malaria Program
IRS: Indoor residual spraying
ITN: Insecticide Treated Nets
VBD: Vector-borne disease
WHO: World Health Organization
LMICs: Low- and/or middle-income countries
GTS: Global Technical Strategy
NSP- ME: National Strategic Plan for Malaria Elimination
KPK: Khyber Pakhtunkhwa
FATA: Federally Administered Tribal Areas
AJK: Azad Jammu and Kashmir
