# Supplementary material for: Malaria vector control strategies in Pakistan: a scoping review
Source: BMC Infect Dis. 2025 Jul 29;25:953. doi: 10.1186/s12879-025-11347-x (PMC12309168; doi:10.1186/s12879-025-11347-x)
Supplement: Supplementary file 1 — Supplementary Material 1: Table 1. Study Characteristics (Included Articles=46). [file 12879_2025_11347_MOESM1_ESM.docx]

| **Supplementary Table 1: Study Characteristics (Included Articles=46)** | | | | |
| --- | --- | --- | --- | --- |
| **Title** | Study Author Last Name | **Year** | **Province** | **Study Design** |
| 1. **A quantitative mosquito survey of 7 villages in Punjab Province, Pakistan with notes on bionomics, sampling methodology and the effects of insecticides.** | Reisen *et al.* [35] | 1978 | Punjab | Cross-sectional |
| 1. **Malaria control and long-term periodicity of the disease in Pakistan.** | de Zulueta *et al.* [36] | 1980 | Punjab | Cross-sectional |
| 1. **A large-scale evaluation of pirimiphos-methyl 25% WP during 1980-1981 for malaria control in Pakistan** | Nasir *et al.* [37] | 1982 | Punjab | Cross-sectional |
| 1. **Population dynamics of some Pakistan mosquitoes: the impact of residual organophosphate insecticide spray on anopheline relative abundance.** | Reisen *et al.* [38] | 1986 | Punjab | Experimental study |
| 1. **Exposure and health assessment during application of lambda-cyhalothrin for malaria vector control in Pakistan.** | Chester *et al.* [39] | 1992 | Punjab | Experimental study |
| 1. **Failure of passive zooprophylaxis: cattle ownership in Pakistan is associated with a higher prevalence of malaria** | Bouma *et al.* [40] | 1995 | KPK | Cross-sectional |
| 1. **Pyrethroid sprayed tents for malaria control: an entomological evaluation in Pakistan** | Hewitt *et al.* [41] | 1995 | KPK | Experimental study |
| 1. **Pyrethroid-impregnated bed nets for personal protection against malaria for Afghan refugees.** | Rowland *et al.* [42] | 1996 | KPK | Experimental study |
| 1. **Self-protection from malaria vectors in Pakistan: an evaluation of popular existing methods and appropriate new techniques in Afghan refugee communities.** | Hewitt *et al.* [43] | 1996 | KPK | Cross-sectional |
| 1. **Chloroquine resistance in Pakistan and the upsurge of falciparum malaria in Pakistani and Afghan refugee populations** | Shah *et al.* [44] | 1997 | KPK | Cross-sectional |
| 1. **An investigation of the relationship between depth to groundwater and malaria prevalence, Punjab, Pakistan** | Donnelly *et al.* [45] | 1997 | Punjab | Cross-sectional |
| 1. **Transmission and control of vivax malaria in Afghan refugee settlements in Pakistan.** | Rowland *et al.* [46] | 1997 | KPK | Cross-sectional |
| 1. **Sustainability of pyrethroid-impregnated bednets for malaria control in Afghan communities.** | Rowland *et al.* [47] | 1997 | KPK | Cross-Sectional |
| 1. **Malaria control in the Afghan refugee camps of western Pakistan** | Rowland *et al.* [48] | 1999 |  | Letter to the editor |
| 1. **Control of zoophilic malaria vectors by applying pyrethroid insecticides to cattle.** | Hewitt *et al.* [49] | 1999 | KPK | Experimental study |
| 1. **Control of malaria in Pakistan by applying deltamethrin insecticide to cattle: a community-randomized trial** | Rowland *et al*[50] | 2001 | KPK | Experimental study |
| 1. **Malaria control in Afghan refugee camps: novel solutions** | Rowland *et al.* [51] | 2001 | KPK | Letter to the Editor |
| 1. **Indoor residual spraying with alphacypermethrin controls malaria in Pakistan: a community-randomized trial** | Rowland *et al.* [52] | 2001 | Punjab | Experimental Study |
| 1. **Comparison of three pyrethroid treatments of top-sheets for malaria control in emergencies: entomological and user acceptance studies in an Afghan refugee camp in Pakistan** | Graham *et al.* [53] | 2002 | KPK | Experimental Study |
| 1. **Adult anopheline ecology and malaria transmission in irrigated areas of South Punjab, Pakistan** | Herrel *et al.* [54] | 2004 | Punjab | Cross-sectional |
| 1. **Tents pretreated with insecticide for malaria control in refugee camps: an entomological evaluation.** | Graham *et al.* [55] | 2004 | KPK | Experimental study |
| 1. **Multicountry field trials comparing wash-resistance of PermaNet and conventional insecticide-treated nets against anopheline and culicine mosquitoes** | Graham *et al.* [56] | 2005 | KPK | Experimental study |
| 1. **Departmental audit of malaria control programme 2001-2005 north west frontier province (NWFP).** | Asif *et al.* [57] | 2008 | KPK | Audit |
| 1. **Epidemic of Plasmodium falciparum malaria involving substandard antimalarial drugs, Pakistan, 2003.** | Leslie *et al.* [58] | 2009 | FATA | Cross-sectional |
| 1. **Pesticide Susceptibility Status of Anopheles Mosquitoes in Four Flood-Affected Districts of South Punjab, Pakistan** | Rathor *et al.* [59] | 2013 | Punjab | Experimental study |
| 1. **Susceptibility/Resistance status of Selected Insecticides in Anopheles Mosquitoes of District Gujrat, Punjab, Pakistan.** | Nazir *et al.* [60] | 2013 | Punjab | Cross-sectional |
| 1. **Prevalence and insecticide resistance status of malaria vectors in Talagang (Punjab), Pakistan.** | Malik *et al.* [61] | 2013 | Punjab | Cross-sectional |
| 1. **Insecticide susceptibility/resistance status of Anopheles mosquitoes in District Bahawalpur, Punjab Pakistan: an entomological survey.** | Mehmood *et al.* [62] | 2013 | Punjab | Cross-sectional |
| 1. **Effect of Tilapia mossembica stocking on larval growth of mosquitoes with special reference to Toaedes Mosquitoes in village ponds of tehsil Yazman, Pakistan** | Haidera *et al.* [63] | 2013 | Punjab | Experimental study |
| 1. **Determination of insecticide susceptibility/resistance status of Anopheline mosquitoes in district Mirpur AJ&K.** | Batool *et al.* [64] | 2014 | AJK | Cross-sectional |
| 1. **Susceptibility and irritability of adult forms of main malaria vectors against insecticides used in the indoor residual sprays in Muzaffargarh District, Pakistan: a field survey.** | Rana *et al.* [65] | 2014 | Punjab | Cross-sectional |
| 1. **Susceptibility/resistance of selected insecticides in Anopheles mosquitoes of district Mirpur Khas, Sindh, Pakistan** | Hammad *et al.* [66] | 2015 | Sindh | Experimental study |
| 1. **Studies on the efficacy of selected insecticides against Anopheles mosquitoes of village Goth Bhoorji (Sindh) Pakistan** | Hammad *et al.* [67] | 2015 | Sindh | Cross-sectional |
| 1. **Mind the gap: residual malaria transmission, veterinary endectocides and livestock as targets for malaria vector control** | Chaccour *et al.* [68] | 2016 | Pakistan | Letter to the Editor |
| 1. **Epidemiology and clinical burden of malaria in the war-torn area, Orakzai Agency in Pakistan** | Karim *et al.* [69] | 2016 | KPK | Cross-sectional |
| 1. **Cost-effectiveness of adding indoor residual spraying to case management in Afghan refugee settlements in Northwest Pakistan during a prolonged malaria epidemic** | Howard *et al.* [70] | 2017 | KPK | Experimental Study |
| 1. **Effects of deltamethrin treated uniform on malaria prophylaxis in troops of Bahawalpur garrison** | Younis *et al.* [71] | 2017 | Punjab | Experimental study |
| 1. **Cost-effectiveness of adding indoor residual spraying to case management in Afghan refugee settlements in Northwest Pakistan during a prolonged malaria epidemic.** | Howard *et al*[72]. | 2017 | KPK | Experimental study |
| 1. **Occurrence and seasonal variation of human Plasmodium infection in Punjab Province, Pakistan.** | Qureshi *et al.* [73] | 2019 | Punjab | Cross-sectional |
| 1. **Malaria epidemiology and comparative reliability of diagnostic tools in Bannu; an endemic malaria focus in south of Khyber Pakhtunkhwa, Pakistan** | Jahan *et al.* [74] | 2019 | KPK | Cross-sectional |
| 1. **Insecticide susceptibility status and major detoxifying enzymes activity in Anopheles subpictus from Kasur, Pakistan** | Naeem *et al.* [75] | 2019 | Punjab | Experimental Study |
| 1. **Susceptibility/resistance status of malaria vector anophelines species in Bajaur agency, FATA** | Farooqi *et al.* [76] | 2019 | FATA | Experimental Study |
| 1. **Baseline survey for malaria prevalence in Khyber Pakhtunkhwa Province, Pakistan.** | Qureshi *et al.* [77] | 2020 | KPK | Cross-sectional |
| 1. **Effectiveness of a health education intervention on the use of long-lasting insecticidal nets for the prevention of malaria in pregnant women of Pakistan: a quasiexperimental study** | Kumar *et al.* [78] | 2020 | Sindh | Experimental study |
| 1. **Prevalence of clinical malaria and household characteristics of patients in tribal districts of Pakistan** | Karim *et al.* [79] | 2021 | KPK | Cross-sectional |
| 1. **Variability in susceptibility status of malaria vectors and other Anopheles species against different insecticides in district Faisalabad, Central Punjab** | Mohsin *et al.* [80] | 2021 | Punjab | Cross-sectional |
